# Sinus tarsi adipose tissue as a neuro-adipose niche in progressive collapsing foot deformity: neural innervation and stem cell properties

**DOI:** 10.3389/fmed.2026.1925286

**Published:** 2026-10-14

**Authors:** Antonio Mazzotti, Alberto Arceri, Cesar de Cesar Netto, Francesca Salamanna, Luca Cavazza, Gabriele Bilancia, Cesare Faldini, Gianluca Giavaresi, Francesca Veronesi

**Affiliations:** 1 1st Orthopaedics and Traumatologic Clinic, IRCCS Istituto Ortopedico Rizzoli, Bologna, Italy; 2 Department of Biomedical and Neuromotor Sciences (DIBINEM), Alma Mater Studiorum University of Bologna, Bologna, Italy; 3 Department of Orthopaedic Surgery, Foot and Ankle Division, Duke University School of Medicine, Durham, NC, United States; 4 Surgical Sciences and Technologies, IRCCS Istituto Ortopedico Rizzoli, Bologna, Italy

**Keywords:** adipose-derived stem cells, age-related differences, neural markers, sex-related differences, sinus tarsi

## Abstract

**Purpose:**

The adipose tissue of the sinus tarsi has traditionally been viewed as a passive structure. Emerging evidence indicates it may function as a biologically active, innervated microenvironment contributing to proprioception and tissue homeostasis. As a rich source of mesenchymal stem cells, it also holds regenerative potential. However, its neural characteristics and stem cell properties remain underexplored.

**Methods:**

Adipose tissue samples were obtained from 20 patients undergoing surgery for symptomatic flatfoot deformity (progressive collapsing foot deformity - PCFD) and stratified by age (<18 vs. ≥ 18 years) and sex. Histology (H&E) and immunohistochemistry (S100, p75, PGP9.5) assessed neural elements, with quantitative analysis of immunopositive areas. Adipose-derived stem cells (ADSCs) were isolated and evaluated for viability (Alamar Blue) and osteogenic differentiation (gene expression: RUNX2, ALPL, COL1A1, BGLAP).

**Results:**

Histology revealed preserved architecture with age-related stromal expansion, more pronounced in males ≥18 years. Neural markers are localized primarily to the stromal/vascular compartment, with p75 predominant across groups (p < 0.0001). Younger females showed highest neural marker expression; significant age-associated decline in patients with PCFD occurred in S100 and p75. ADSCs from younger females exhibited superior viability and early osteogenic marker expression, while aging correlated with reduced proliferation and a shift toward mature osteogenic phenotype, especially in males.

**Conclusion:**

The sinus tarsi harbors a dynamic neuro-adipose niche with age- and sex-dependent neural and stem cell features. This tissue may link proprioception with regenerative capacity and represents a promising autologous cell source for foot and ankle applications.

## 1 Introduction

The sinus tarsi is an osseoligamentous recess located between the talus and calcaneus, bounded anteriorly by the talonavicular joint and posteriorly by the posterior facet of the subtalar joint, with medial extension into the tarsal canal. This region contains several critical structures, including a substantial amount of periarticular adipose tissue (1).

Adipose tissue, traditionally regarded as an energy storage organ, is now recognized as a metabolically active and highly innervated tissue with endocrine, thermogenic, and sensory functions (2). Both white and brown adipose tissues receive sympathetic and sensory innervation capable of responding to mechanical and metabolic stimuli (3–5). Immunohistochemical studies have demonstrated nerve fibers within periarticular adipose tissue associated with musculoskeletal structures, suggesting roles in mechanosensation and pain perception. These findings support the concept that periarticular adipose tissue actively contributes to proprioception and joint homeostasis (6, 7).

Proprioceptive input arises from mechanoreceptors located in periarticular soft tissues and is conveyed to the central nervous system to provide information on joint position, movement, and loading (8). This sensory feedback contributes to postural control, coordination, and protective reflexes, enabling joint stabilization during static and dynamic activities (9). According to the classification originally proposed by Freeman and Wyke and later modified by Hagert, mechanoreceptors include Ruffini corpuscles, Pacinian corpuscles, Golgi-Mazzoni corpuscles, free nerve endings, and unclassified endings (10, 11). The density and distribution of these mechanoreceptors are key determinants of proprioceptive acuity (12–14).

Alterations in mechanoreceptor density and function have been investigated in musculoskeletal disorders, particularly osteoarthritis (OA), where reduced proprioceptive accuracy may contribute to onset and progression (12). Decreased mechanoreceptor numbers have been reported in cruciate ligaments and other periarticular structures, associated with worse clinical outcomes (15–20). Despite increasing evidence of neural elements within the sinus tarsi (11, 21–23), detailed investigations of its adipose tissue innervation remain limited. To date, only one cadaveric study has demonstrated nerve endings interspersed among adipocytes, suggesting a potential role in proprioception and nociception (1).

Beyond its sensory role, adipose tissue serves as a major reservoir of mesenchymal stem cells (MSCs), multipotent progenitors capable of differentiating into connective tissue lineages and exerting trophic and immunomodulatory effects (24). Adipose-derived stem cells (ADSCs) have emerged as a practical source for musculoskeletal regeneration due to accessibility, high yield, and preserved differentiation potential (25–27). Neurogenic signaling and mechanical stimuli regulate stem cell behavior, supporting the concept of a functional neuro-adipose niche (28).

No prior studies have investigated ADSCs derived specifically from sinus tarsi adipose tissue or explored relationships between its neural components and stem cell potential. Given its anatomical accessibility during foot and ankle surgery, this tissue may represent a clinically relevant and previously underexplored source of regenerative cells. The primary aim of this pilot study was to evaluate the presence and distribution of neural elements within sinus tarsi adipose tissue from patients with symptomatic flatfoot deformity (progressive collapsing foot deformity – PCFD) (29, 30) and to assess the regenerative potential of resident ADSCs, with correlation to age and sex. We hypothesized that the adipose tissue of the sinus tarsi in patients PCFD represents a neuro-adipose microenvironment characterized by the coexistence of detectable neural elements within the stromal compartment and resident ADSCs with osteogenic differentiation potential, both of which may vary according to age and sex. Specifically, we anticipated higher neural marker expression and greater stromal cell viability and early osteogenic commitment in younger patients, particularly females.

## 2 Materials and methods

The study was approved by the local Ethics Committee of Area Vasta Emilia Centro (Protocol n. CE-AVEC: 784/2023/Sper/IOR), and written informed consent was obtained from all participants prior to enrollment. Patients aged between 8 and 90 years undergoing surgical treatment for PCFD were eligible for inclusion. Surgical procedures included subtalar arthroereisis with endorthotic (calcaneal stop) implants or subtalar arthrodesis. An adipose tissue volume of at least 1 cm³ within the sinus tarsi region from each patient was required for inclusion. Exclusion criteria comprised the presence of active infections at the time of surgery, pregnancy, contraindications to surgical treatment, and autoimmune diseases.

Adipose tissue samples, otherwise discarded during surgery, were collected under sterile conditions in the operating room and immediately transferred to the laboratory. Under aseptic conditions, each sample was divided into two portions: one designated for histological and immunohistochemical analyses, and the other for the isolation and biological and molecular characterization of adipose-derived stem cells (ADSCs).

### 2.1 Histological and immunohistochemical evaluations

A portion of the adipose tissue was fixed in formalin, dehydrated in graded alcohol solutions, cleared in xylene, and embedded in paraffin. Histological sections (5 ± 1 µm thick) were obtained using a semiautomated microtome (Microm H340E, Heidelberg, Germany). Three sections per sample were stained with hematoxylin and eosin (H&E; Sigma-Aldrich) to assess tissue morphology. Three additional sections were used for immunohistochemical analysis of neural markers. Sections were dewaxed in decreasing graded ethanol solutions, rinsed in phosphate-buffered saline (PBS) for 10 min, and immunostained for S100, p75, and PGP9.5 using primary antibodies (Proteintech Group, Inc., Rosemont, IL, USA). After antigen unmasking with 0.2% Pronase (Sigma–Aldrich) for 30 min at 37 °C and blocking with Blocking Serum (Vectastain Universal Quick Kit, Vector Laboratories), sections were incubated overnight at 4 °C with rabbit polyclonal antibodies against S100, p75, and PGP9.5. After rinsing, sections were incubated with anti-rabbit HRP-conjugated secondary antibody (Bethyl Laboratories) and Streptavidin/Peroxidase complex, and the reaction was developed using Vector NovaRed Substrate Kit (Vector Laboratories). Negative controls were performed by omitting the primary antibody. Histological and immunohistochemical images were acquired with a digital pathology slide scanner (Aperio ScanScope CS, Aperio Technologies, Leica Biosystems, Milan, Italy). The expression of S100, p75, and PGP9.5 proteins was quantified as the percentage of immunopositive area relative to the total region of interest (ROI) using Aperio ImageScope software (version 12.4.6.5003).

### 2.2 Biological and molecular evaluations

The remaining portion of adipose tissue was enzymatically digested with 0.075% collagenase II (Sigma Aldrich, St. Louis, MO, USA). The enzymatic reaction was stopped by the addition of complete Dulbecco’s modified Eagle’s medium (DMEM) supplemented with 10% fetal bovine serum (FBS) (Lonza, Verviers, Belgium), 100 U/mL penicillin, 100 mg/mL streptomycin (Gibco, Invitrogen), and 5 mg/mL plasmocin (Invivogen, San Diego, CA, USA). Nucleated cells were centrifuged and seeded in complete medium in culture flasks. At subconfluence (passage 2), adherent cells were detached and seeded in 24-well plates at a density of 5 × 10³ cells/cm². For osteogenic differentiation, the medium was supplemented with 50 µg/mL ascorbic acid 2-phosphate, 7 mM β-glycerophosphate, and 10⁻⁷ M dexamethasone for 21 days.

Cell viability was evaluated after 1 and 3 weeks of culture using the Alamar Blue assay (Thermo Fisher Scientific). A 1:10 mixture of dye and fresh culture medium was added to each well, incubated for 4 h at 37 °C, and fluorescence was measured at 530 nm excitation/590 nm emission using a microplate reader (VICTOR X2030, Perkin Elmer). Results were expressed as relative fluorescence units (RFUs). Osteogenic differentiation was further assessed by Alizarin Red S staining (2%) to detect calcium deposits after 21 days in osteogenic medium.

Osteogenic gene expression was evaluated by quantitative real-time PCR (qRT-PCR). Total RNA was isolated using TRIzol reagent (Ambion, Life Technologies) followed by purification with the PureLink RNA Mini Kit (Ambion). RNA concentration was quantified using a NanoDrop 2000 spectrophotometer (Thermo Scientific). Reverse transcription was performed with the SuperScript VILO cDNA Synthesis Kit (Life Technologies). Gene expression of RUNX2, ALPL, COL1A1, and BGLAP was assessed using SYBR Green PCR Master Mix (Qiagen) on a LightCycler 2.0 Instrument (Roche Diagnostics). The protocol included an initial denaturation at 95 °C for 15 min, followed by 25–40 cycles of amplification (95 °C for 15 s, annealing at 55 °C for 20 s, and extension at 72 °C for 20 s). Relative expression was calculated using the 2^−*ΔΔ*Ct method with GAPDH as the reference gene (31). Primer sequences, amplicon lengths, and annealing temperatures are reported in Table 1.

**Table 1:** Sequence of primers, amplicon length, and annealing temperatures used for the evaluation of gene expression by qPCR.

Gene	Primer forward (5′→3′)	Primer reverse (5′→3′)	Amplicon lenght	Annealing temperature
GAPDH	QuantiTect Primer Assay (Qiagen) Hs_GAPDH_1_SG		95 bp	55 °C 20''
ALPL	QuantiTect Primer Assay (Qiagen) Hs_ALPL_1_SG		110 bp	55 °C 20''
BGLAP	QuantiTect Primer Assay (Qiagen) Hs_BGLAP_1_SG		90 bp	55 °C 20''
COL1A1	QuantiTect Primer Assay (Qiagen) Hs_COL1A1_1_SG		118 bp	55 °C 20''
RUNX2	QuantiTect Primer Assay (Qiagen) Hs_RUNX2_1_SG		101 bp	55 °C 20''

### 2.3 Sample size and statistics

This pilot study included adipose tissue samples from 20 patients (*n* = 5 per group), stratified into four groups based on age (<18 years versus ≥18 years) and sex (male versus female). The pilot sample represented approximately 20% of the total planned cohort for the larger ongoing project. The sample size was calculated using G*Power software (version 3.1.9.7, Kiel University, Germany), based on a factorial design including age group and sex, assuming a power of 1–β = 0.80, a significance level α = 0.05, and a medium-to-large effect size (f ≥ 0.40). The present pilot cohort was not intended to provide definitive statistical power or population-level generalizability, but rather to generate preliminary biological data, estimate variability and effect sizes, and inform the design of the larger study. Accordingly, the findings of the present pilot analysis should be considered exploratory and hypothesis-generating.

All statistical analyses were performed using R software (version 4.3.2, R Core Team, Vienna, Austria). Data are presented as mean ± standard deviation (SD) for normally distributed variables or median with interquartile range [IQR] for non-normal data. Normality of distribution was assessed using the Shapiro–Wilk test, and homogeneity of variances was evaluated with Levene’s test.

For inferential analyses, two-way analysis of variance (ANOVA) or linear mixed-effects models were used to evaluate the main effects of age group, sex, and their interaction on immunohistochemical positivity rates and ADSC outcomes (viability and gene expression), with time as a repeated measure where appropriate. When significant interactions or main effects were detected, *post-hoc* pairwise comparisons were performed using Bonferroni or Tukey adjustments for multiple comparisons. For data that violated normality or homogeneity of variance assumptions, nonparametric alternatives such as the Kruskal–Wallis test followed by Dunn’s *post-hoc* test or the Aligned Rank Transform procedure were applied. A two-tailed *p*-value < 0.05 was considered statistically significant. All analyses were conducted in a blinded manner with respect to sample group allocation whenever feasible.

## 3 Results

The demographic characteristics of the 20 patients (10 females and 10 males) are summarized in Table 2. In both sexes, patients were evenly distributed by age group, with five individuals younger than 18 years and five aged 18 years or older. As expected, significant age differences existed between the <18-year and ≥18-year groups in both females (*p* = 0.0058) and males (*p* = 0.0012). Within the ≥18-year group, males were significantly older than females (*p* = 0.0217).

**Table 2:** Demographic characteristics of patients.

**Age (Years)**	**Females (*n* = 10)**	**Males (*n* = 10)**	**p-value (Females vs Males)**
**< 18 (*n* = 10)**	10.60 ± 1.67	12.60 ± 1.52	*p* = 0.0833
**≥ 18 (*n* = 10)**	37.80 ± 11.56	60.40 ± 13.39	***p* = 0.0217** *
(< 18 vs ≥ 18)	***p* = 0.0058** *	***p* = 0.0012** *	

* bolded values when *p* < 0.05.

### 3.1 Histological and immunohistochemical evaluations

H&E staining demonstrated preserved lobular architecture of the adipose tissue in all groups, characterized by unilocular adipocytes with clear cytoplasm and peripherally located nuclei (Figure 1). In subjects younger than 18 years, both females and males exhibited a homogeneous adipocyte distribution with thin and delicate fibrous septa and minimal stromal components. No appreciable differences were observed between sexes in this age group. In contrast, samples from subjects aged ≥18 years displayed more heterogeneous morphology, with increased thickness of fibrous septa and areas of stromal expansion. These changes were more pronounced in males, who showed denser connective tissue, more evident vascular structures, and occasional small nerve bundles compared with females. No significant inflammatory infiltrate or cellular atypia was observed in any group.

**Figure 1:**
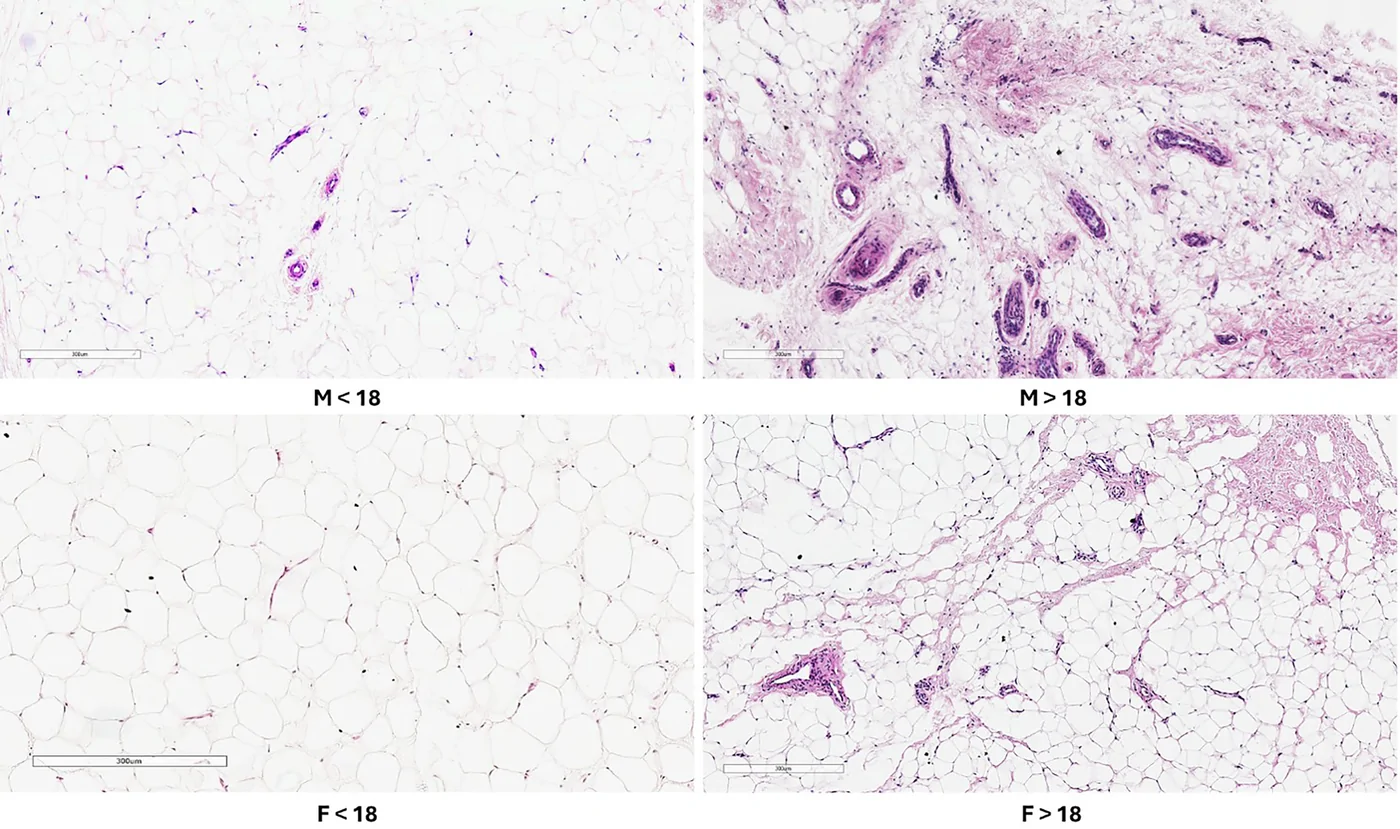
Representative H&E-stained sections of adipose tissue from subjects stratified by age (<18 and ≥18 years) and sex (females, F; males, M). Scale bar: 300 µm. Magnification: 10×.

Immunohistochemical analysis revealed the presence of S100-, p75-, and PGP9.5-positive structures primarily within the stromal compartment, often localized in nerve bundles along fibrous septa and in association with vascular elements (Figure 2). Qualitatively, immunoreactivity appeared more organized and abundant in subjects aged ≥18 years, particularly in males.

**Figure 2:**
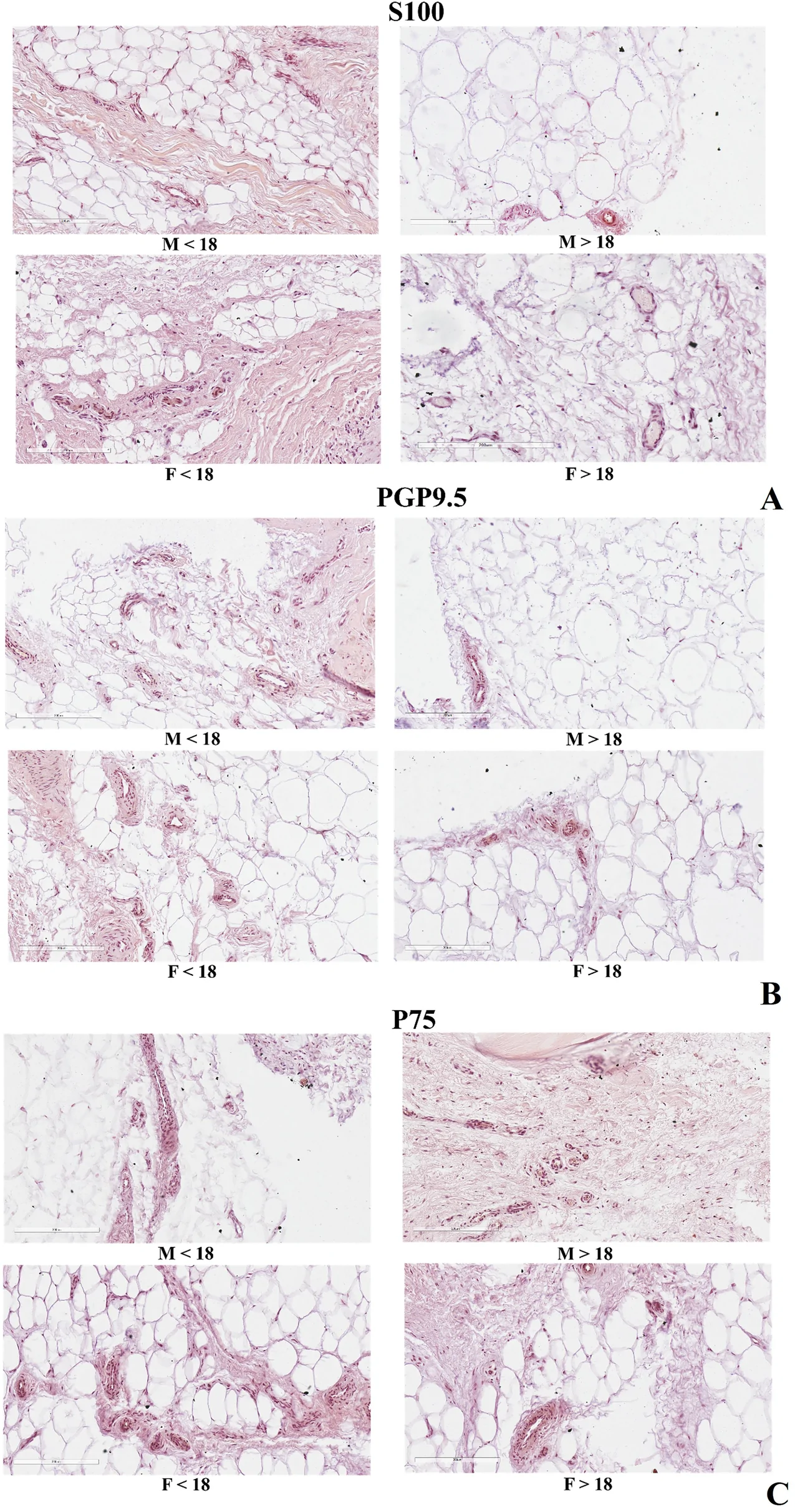
Immunohistochemical staining for neural markers in sinus tarsi adipose tissue. Representative images for **(A)** S100, **(B)** p75, and **(C)** PGP9.5 from subjects stratified by age and sex. Scale bar: 200 µm. Original magnification: 10×.

Quantitative analysis supported these observations and revealed distinct patterns of marker expression according to both sex and age. Immunohistochemical staining revealed significant differences in marker expression among S100, PGP9.5, and p75 across all groups (Figure 3). In females under 18 years of age, p75 expression was significantly higher than S100 expression (4.7% [2.8, 6.8]) and PGP9.5 (11.0% [9.0, 13.0]) (*p* < 0.0005). Furthermore, in females under 18 years of age, S100 expression was significantly higher than PGP9.5 (6.2% [4.2, 8.3], *p* < 0.0005), whereas this difference was no longer observed in older females. Among females over 18 years of age, p75 levels remained significantly higher (*p* < 0.0005) than both S100 (10.4% [8.4, 12.4]) and PGP9.5 (10.8% [8.8,12.9]) levels, whereas S100 and PGP9.5 did not differ significantly from each other.

**Figure 3:**
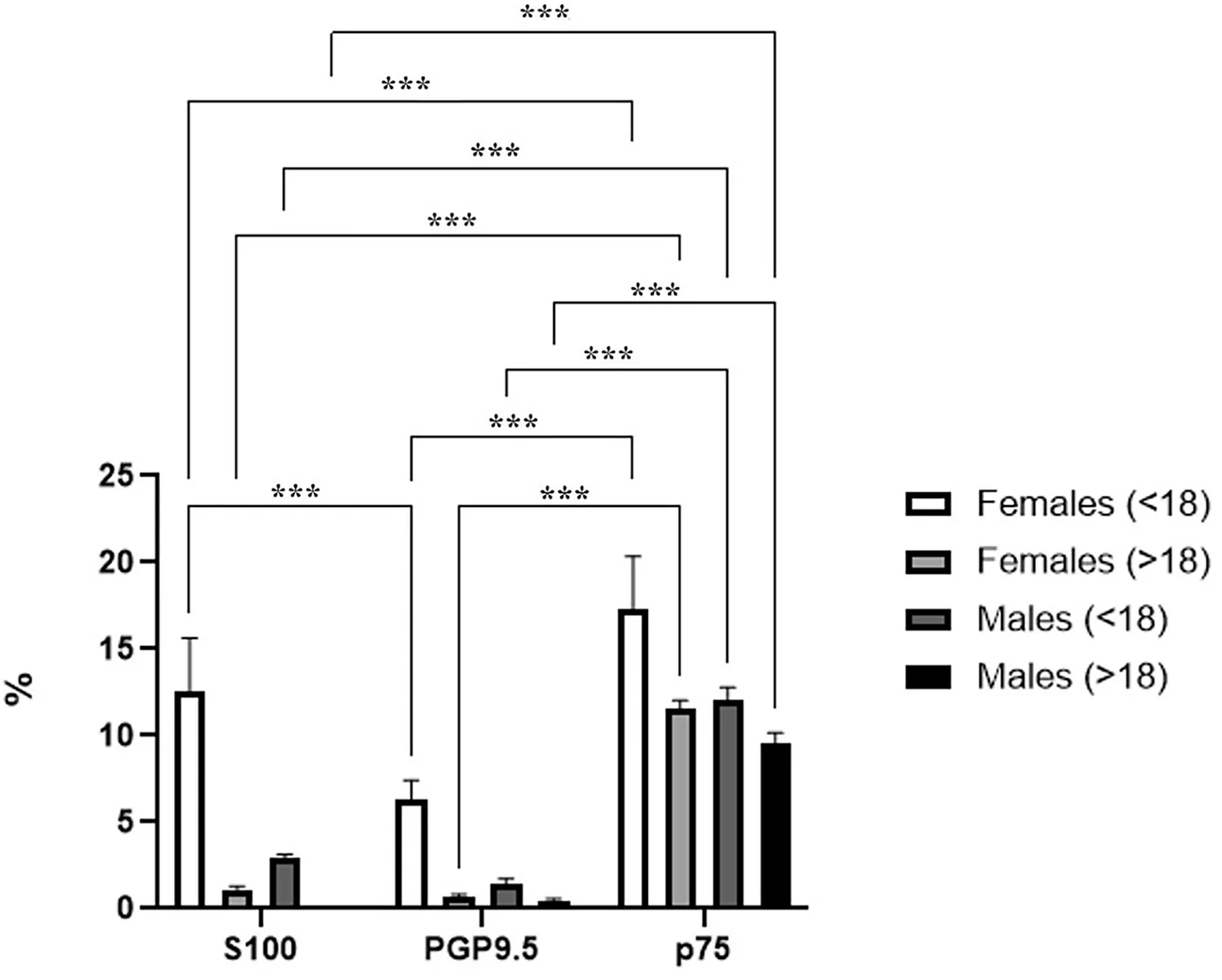
Immunohistochemical positivity rates (%) for S100, PGP9.5, and p75 stratified by sex and age group. Two-way ANOVA (age-sex interaction *F* = 7.49, *p* < 0.0005). Multiple comparisons: *, *p* < 0.05; **, *p* < 0.005; ***, *p* < 0.0005.

A comparable pattern was observed in males under 18 years, in whom p75 expression was significantly higher (*p* < 0.0005) than both S100 (9.1% [7.1, 11.2) and PGP9.5 (10.7% [8.7, 12.7]) expression. A similar trend was also observed in males over 18 years of age (p75 vs. S100: 9.5% [7.5, 11.5]; p75 vs. PGP9.5: 9.2% [7.2,11.2]), with no significant difference between S100 and PGP9.5 expression in either male group. These findings indicate that p75 represents the predominant neural marker in tarsal sinus adipose tissue across all groups, while age- and sex-related variations predominantly affect S100 and PGP9.5 expression.

To further investigate the effects of age and sex on neural marker expression, pairwise comparisons between groups were performed (Figure 4). When comparing sexes within age groups, females under 18 years showed significantly higher immunohistochemical positivity (*p* < 0.0005) for S100 (9.7% [7.6–11.7]), PGP9.5 (5.0% [3.9–6.0]), and p75 (5.3% [3.2–7.4]) compared to age-matched males. In contrast, no significant sex-related differences were observed in the over-18 group.

**Figure 4:**
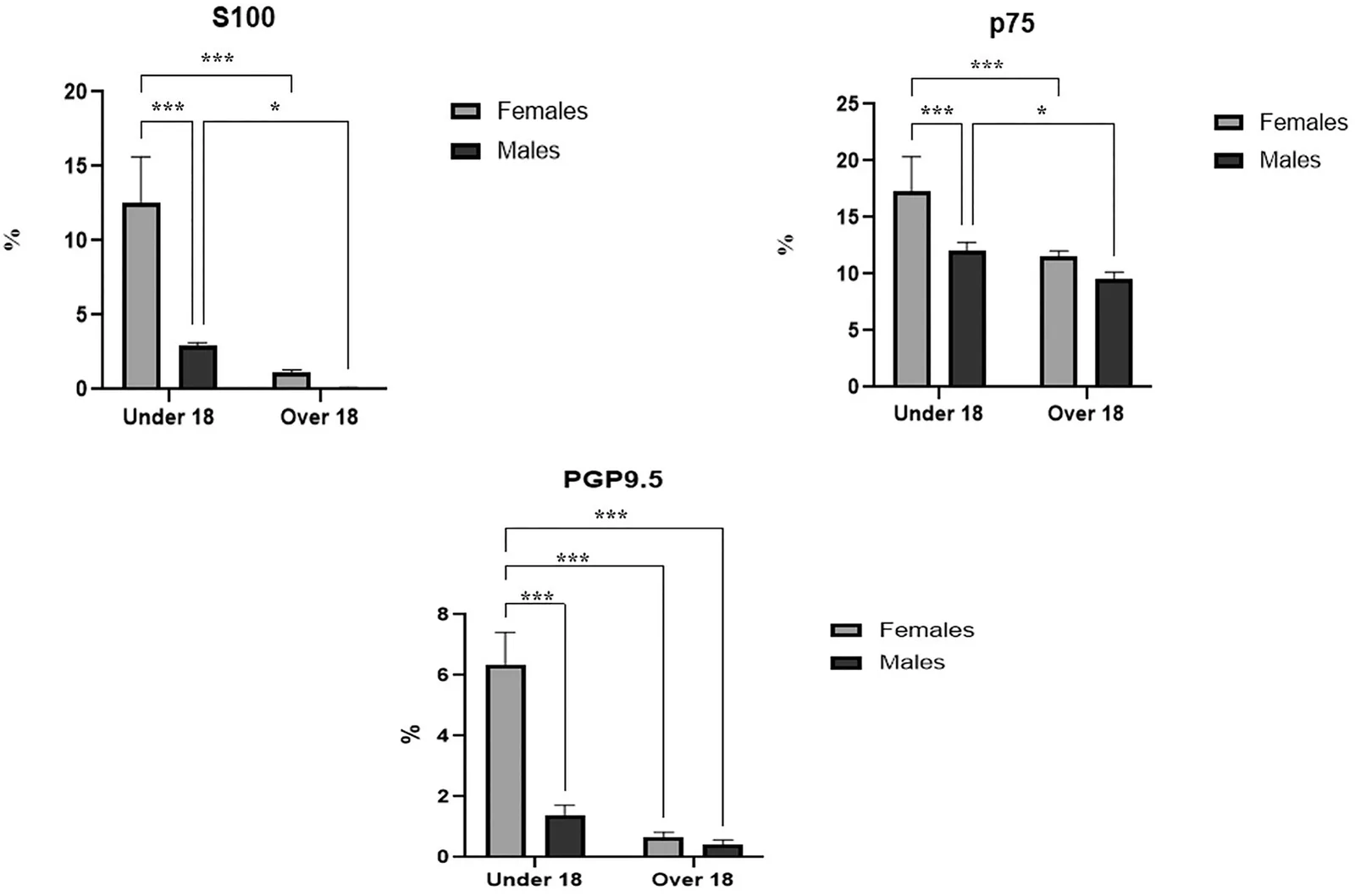
Immunohistochemical positivity rates for S100 (age-sex interaction *F* = 39.7, *p* < 0.0005), PGP9.5 (age-sex interaction *F* = 86, *p* < 0.0005), and p75 (age-sex interaction *F* = 5.62, *p* = 0.031) stratified by sex and age (< 18 vs ≥ 18 years). Multiple comparisons: *, *p* < 0.05; **, *p* < 0.005; ***, *p* < 0.0005.

Within-sex comparisons by age revealed that females over 18 years of age exhibited a significantly lower expression (*p* < 0.0005) of all three markers compared with younger females: S100 (−11.5% [−13.5, −9.4]), PGP9.5 (−5.7% [−6.7, −4.7]), and p75 (−5.8% [−8.0, −3.7]). Similarly, older males showed significant age-related decreases in S100 (−2.8% [−4.9, −0.7]; *p* = 0.011) and p75 (−2.5% [−4.6, −0.3]; *p* = 0.026) expression, while PGP9.5 did not reach statistical significance (Figure 4).

### 3.2 Biological and molecular evaluations

ADSCs exhibited clear age- and sex-related differences in cell viability and osteogenic gene expression.

As regards viability, the mixed effects model revealed significant main effects of sex, age, and time, as well as a significant Sex×Age interaction (all *p* < 0.01), indicating that levels differed across demographic groups and evolved over time in a non-uniform manner (Figure 5). The effect of time was particularly strong, with higher viability at 3 weeks compared to 1 week across all groups.

**Figure 5:**
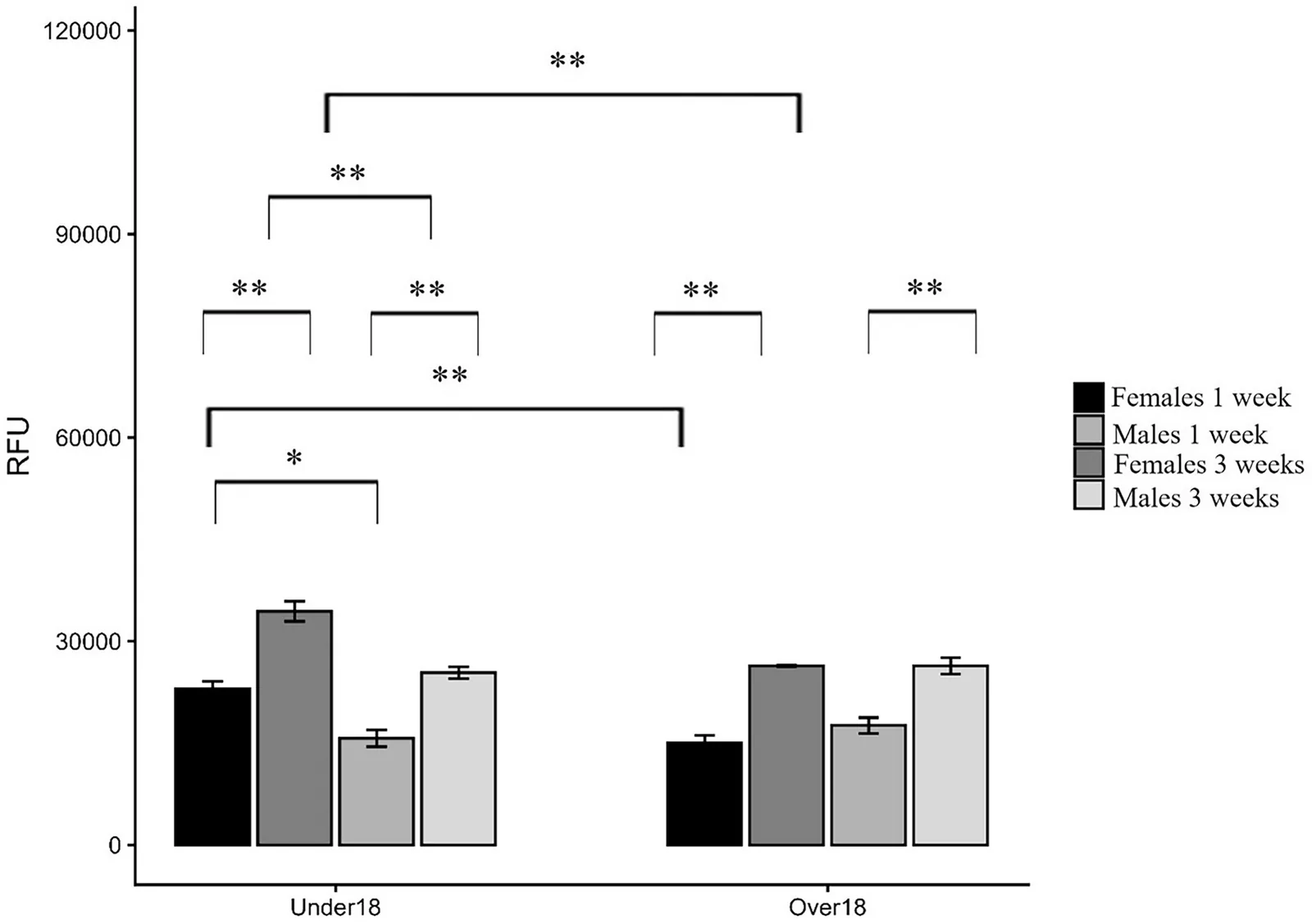
ADSC viability across sex, age, and time. Bars represent estimated marginal means (± SE) from the mixed-model analysis for females and males in the Under-18 and Over-18 groups at 1 week and 3 weeks. Under-18 females showed the highest viability at both time points, with a pronounced increase at 3 weeks. Significant pairwise differences between groups are indicated by brackets and asterisks (* *p* < 0.05, ** *p* < 0.01, *** *p* < 0.001), highlighting strong effects of age, sex, and time on viability.

Post hoc comparisons showed that within each age group, both sexes showed a significant increase from 1 to 3 weeks (all *p* < 0.003). At 1 week, females under 18 years exhibited significantly higher proliferative activity compared with male under 18 years (*p* = 0.012) and females over 18 years (*p* = 0.005). Overall, the under 18 cohort, particularly females, displayed the highest viability over time.

Following 3 weeks of osteogenic induction, the analysis of osteogenic markers revealed distinct patterns across groups (Figure 6).

**Figure 6:**
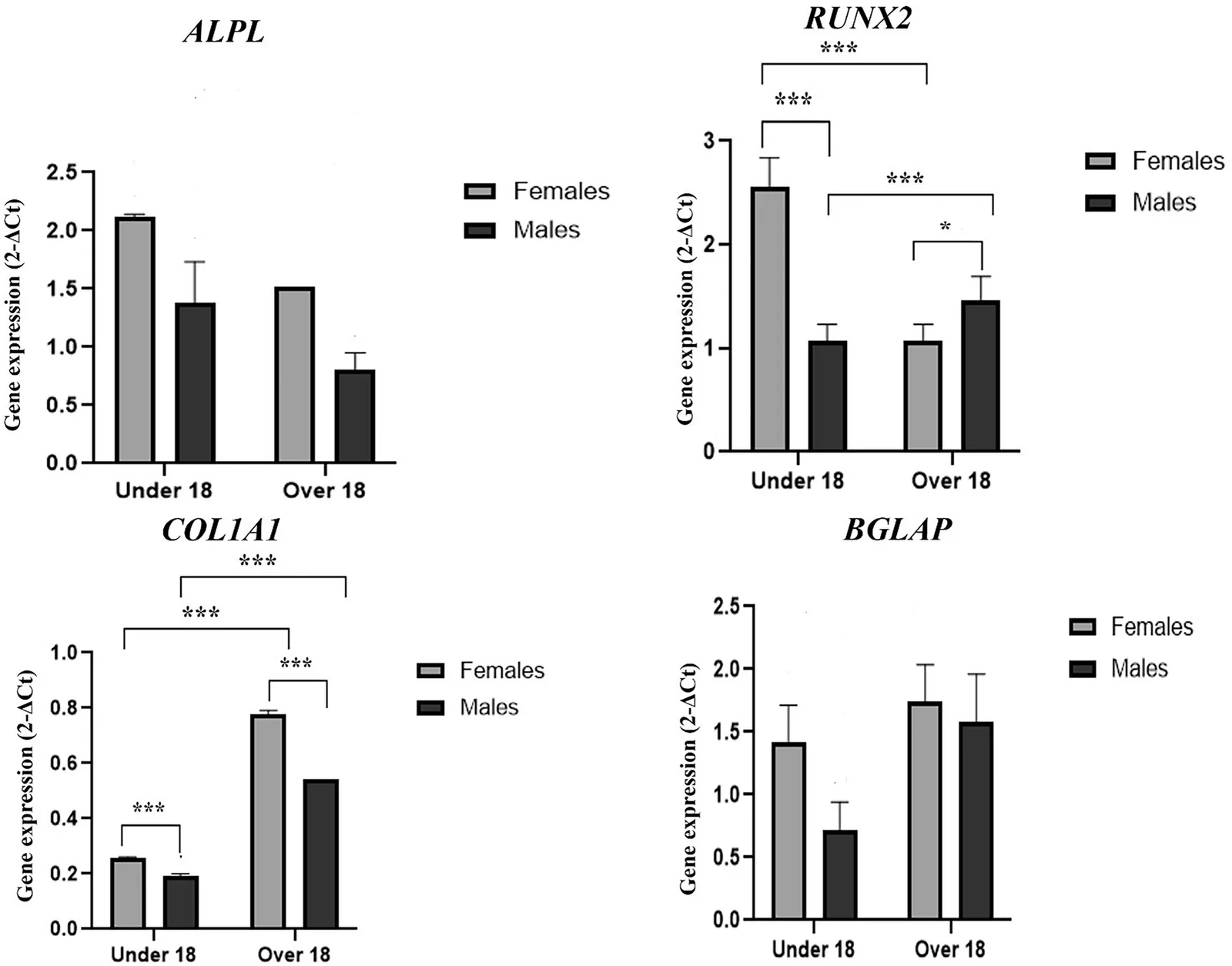
Gene expression levels of ALPL, RUNX2, COL1A1, and BGLAP stratified by sex and age groups (<18 vs. ≥18 years). Two-way RM ANOVA showed age and sex effects for ALPL (age: F = 33.3, *p* < 0.0005; sex: F = 125.2, *p* < 0.0005) and BGLAP (age: *F* = 18.5, *p* = 0.003; sex: *F* = 10.6, *p* = 0.012), while RUNX2 and COL1A1 showed age × sex interaction (RUNX2: *F* = 83.3, *p* < 0.0005; COL1A1: *F* = 723, *p* < 0.0001). Multiple comparisons: *p* < 0.05, **: *p* < 0.005, ***: *p* < 0.0005.

To further investigate the effects of age and sex on osteogenic marker expression, pairwise comparisons between groups were performed where appropriate (Figure 6).

ALPL expression was higher in females than in males overall, with a mean difference of 0.730 [0.580–0.880] and was lower in donors over 18 years compared with younger donors, with a mean difference of −0.590 [−0.825, −0.355] (Figure 6).

For RUNX2 expression, females under 18 years showed higher expression than age-matched males (1.49 [1.20–1.78]; *p* < 0.0005). In the over-18 group, RUNX2 expression was higher in males than in females (0.393 [0.106–0.680]; *p* = 0.011). Within-sex comparisons showed that RUNX2 expression decreased in females over 18 years compared with younger females (−1.49 [−1.83, −1.15]; *p* < 0.0005), whereas males showed an increase with age (0.390 [0.054–0.726]; *p* = 0.028) (Figure 6). COL1A1 expression was higher in females than in males both in donors under 18 years (0.0667 [0.0538–0.0795]; *p* < 0.0001) and in donors over 18 years (0.237 [0.224–0.250]; *p* < 0.0005). Within-sex comparisons showed increased COL1A1 expression in donors over 18 years in both females (0.520 [0.510–0.530]; *p* < 0.0005) and males (0.350 [0.340–0.360]; *p* < 0.0005) (Figure 6). BGLAP expression was higher in females than in males overall, with a mean difference of 0.437 [0.128–0.746] and was higher in donors over 18 years compared with younger donors, with a mean difference of 0.590 [0.274–0.906] (Figure 6).

Collectively, these data indicate that female donors displayed higher expression of selected osteogenic markers, including ALPL and BGLAP, while age was associated with reduced ALPL expression and increased BGLAP expression. However, several statistically supported differences corresponded to relatively modest fold changes, below or close to the commonly used 1.5–2-fold threshold for biological relevance. Therefore, these findings should be interpreted as evidence of subtle transcriptional modulation rather than strong biologically meaningful regulation, with the most pronounced changes observed for RUNX2 in younger females and for the age-related increase of COL1A1 expression in both sexes during osteogenic differentiation.

## 4 Discussion

The present pilot study provides novel insights into the biological complexity of sinus tarsi adipose tissue, demonstrating the coexistence of neural elements and resident stromal cells within this anatomical compartment, together with age- and sex-related variations in neural marker expression and stromal cell behavior. These findings support the characterization of sinus tarsi adipose tissue as a biologically active neuro-adipose microenvironment, while the functional relationships between its neural and stromal components remain to be established. Furthermore, given the small sample size, these findings should be interpreted as preliminary differences across age-sex groups rather than definitive age- or sex-dependent effects.

Traditionally considered a passive filler, adipose tissue within the sinus tarsi emerges from our findings as an active and highly specialized microenvironment, where neural elements and stromal components coexist and potentially interact (11, 21, 32). The consistent predominance of p75 expression across all groups suggests that this tissue is enriched in Schwann cell–associated and neuro-supportive elements rather than mature neuronal structures (33). This is further supported by the relatively lower expression of PGP9.5, a marker of differentiated nerve fibers, indicating that the neural component of this adipose tissue may be characterized by plasticity and regenerative potential rather than stable innervation (34–36).

The biomechanical and proprioceptive relevance of these findings is noteworthy. Cadaveric studies have demonstrated a rich distribution of sensory nerve endings, including mechanoreceptors, within the sinus tarsi fat pad, supporting its established role in monitoring subtalar joint position and motion (1, 21). In the setting PCFD, chronic mechanical overload and subtalar instability likely amplify stress on this neuro-adipose unit, which may contribute to the frequent occurrence of sinus tarsi syndrome and perceived instability. Our observation of organized neural structures within the stromal compartment, along with age-associated decline in patients with PCFD, provides histological support for this proprioceptive function and suggests that long-standing deformity drives adaptive or maladaptive remodeling.

From a pathophysiological perspective, this pattern is particularly relevant in the context of PCFD, where the sinus tarsi is subjected to chronic mechanical stress resultant from subtalar joint instability, and the presence of sinus tarsi and subfibular impingement (staged as Class D PCFD, Peritalar Subluxation) (23, 30, 32, 37, 38). Under these conditions, the increased expression of p75 may reflect an adaptive or reparative response driven by Schwann cells and stromal elements, aimed at maintaining neural integrity and mechanosensory function. Conversely, the reduced expression of PGP9.5 may indicate partial degeneration or remodeling of mature nerve fibers (39, 40).

A key finding of this study is the age-dependent decline in neural marker expression, particularly evident for S100 and p75, which was more pronounced in females. The reduction during aging likely reflects a progressive loss of neuro-supportive capacity and Schwann cell activity with aging, in line with previous evidence describing impaired peripheral nerve regeneration and reduced neurotrophic signaling in older tissues (41–43).

Importantly, these neural changes occurred in parallel with the differences in the biological behavior of ADSCs isolated from the same tissue. Younger female donors, characterized by higher neural marker expression, also exhibited enhanced cell viability and expression of selected early osteogenic markers. This parallel pattern suggests a potential association between neural and stromal cell features within the sinus tarsi microenvironment; however, the present descriptive study does not establish a direct functional or causal relationship between these compartments (44). Neural-derived signals, including neurotrophins and mechanical stimuli, are known to modulate stem cell proliferation and differentiation (45), providing a biological rationale for further investigation of this potential interaction. Dedicated mechanistic studies will therefore be required to determine whether neural elements directly influence the behavior and regenerative properties of resident stromal cells in sinus tarsi adipose tissue.

Consistent with this pattern, aging was associated with both reduced neural marker expression and decreased ADSC viability, further indicating parallel age-related changes in the neural and stromal components of sinus tarsi adipose tissue. Interestingly, while early osteogenic markers (ALPL and RUNX2) decreased with age, the increased expression of matrix-related genes such as COL1A1 suggests a shift toward a more mature but less proliferative phenotype. However, given the exploratory nature of the study and the limited sample size, these findings should be interpreted as preliminary age- and sex-associated patterns rather than definitive differentiation trajectories (25–27).

In the present study, the differentiation potential of ADSCs was specifically investigated toward the osteogenic lineage, while chondrogenic differentiation was not explored. This choice was driven by both biological and clinical considerations. Flatfoot deformity is characterized by altered joint mechanics and progressive structural impairment, which in advanced stages often require surgical interventions such as arthrodesis, where bone formation and successful fusion represent the primary therapeutic objectives (46). Therefore, focusing on osteogenic differentiation provides the most clinically relevant assessment of the regenerative potential of ADSCs derived from sinus tarsi adipose tissue in this context.

Compared with other adipose depots, the sinus tarsi fat pad may offer unique advantages as a stem cell source. Its local conditioning within a mechanically demanding, proprioceptively rich environment could prime resident ADSCs toward musculoskeletal lineages. This local adaptation, combined with the tissue’s routine accessibility during standard flatfoot procedures, positions sinus tarsi-derived ADSCs as a promising minimally invasive autologous option for augmenting ligament reconstructions, arthrodesis, or biologics-enhanced repairs in PCFD. However, the presence of neural elements within sinus tarsi adipose tissue should also be considered when contemplating its use as a local autologous cell source. Given the potential sensory and proprioceptive role of this tissue, harvesting should be performed conservatively to minimize disruption of its neural components and preserve local function. Future studies should determine whether the amount of tissue that can be safely harvested can be quantitatively defined. At the same time, its anatomical proximity to foot and ankle structures may make sinus tarsi adipose tissue an attractive local source of stromal cells for regenerative strategies targeting adjacent tissues.

Another relevant observation is the apparent sexual dimorphism identified in both neural and stem cell-related parameters. Female subjects under 18 years showed the highest expression of neural markers together with superior ADSC viability and early osteogenic commitment. These findings may be partially influenced by hormonal regulation, as estrogens have been shown to modulate peripheral nerve regeneration, angiogenesis, and mesenchymal stem cell metabolism (25–27).

From a regenerative medicine perspective, the identification of ADSCs within sinus tarsi adipose tissue may have practical implications extending beyond flatfoot surgery. In recent years, increasing attention has been directed toward the use of locally harvested adipose-derived cells for orthopedic applications, including bone healing, tendon regeneration, cartilage repair, and treatment of degenerative foot and ankle disorders (24–27). Harvesting tissue from the surgical field itself could reduce donor site morbidity and simplify intraoperative cell procurement procedures.

Several limitations of the present pilot study should be acknowledged. The main limitation of this study is its small sample size (*n* = 5 per group), which limits statistical power and external validity and precludes generalization of the findings to the broader population. The present results should therefore be considered preliminary and hypothesis-generating. The pilot design was intended to provide initial estimates of biological variability and effect sizes to inform the ongoing larger study. Confirmation in a larger, adequately powered cohort will be necessary before drawing definitive conclusions regarding the independent effects of age and sex on the biological characteristics of sinus tarsi adipose tissue. In particular, the adult male group included older subjects compared with females, which may have partially influenced some age-related differences. Second, only a limited panel of neural markers was investigated, and no ultrastructural or functional assessment of mechanoreceptors was performed. Third, although adherent cells were isolated from sinus tarsi adipose tissue and evaluated for viability and osteogenic differentiation, a comprehensive phenotypic characterization according to ISCT criteria was not performed. Therefore, the cellular findings should be interpreted within the limits of the characterization performed in the present study. In addition, the biological characterization focused on the osteogenic lineage and did not include other differentiation capacities or regenerative properties, such as immunomodulatory activity, angiogenic potential, or secretome composition. Future studies incorporating a complete immunophenotypic characterization and broader functional assessment will be required to more comprehensively define the properties of the resident stromal cell population. Finally, the study lacked healthy control sinus tarsi adipose tissue. Sinus tarsi adipose tissue could only be ethically collected from patients undergoing surgery for PCFD, as obtaining comparable tissue from healthy individuals would have required an additional invasive procedure solely for research purposes. Therefore, the present design does not allow us to distinguish whether the observed differences are attributable to physiological aging, PCFD-associated pathological changes, or their interaction. The age- and sex-stratified comparisons should consequently be interpreted as exploratory within the PCFD population. An additional limitation concerns the unequal age distribution between sexes within the adult subgroup, with female participants being younger than male participants. Although age group and sex were explicitly considered in the statistical analysis, the small sample size prevented robust adjustment for age as a continuous variable and for potential age-related hormonal factors. Menopausal status and circulating hormone levels were not systematically available and therefore could not be incorporated into the analysis. Consequently, the observed sex- and age-associated differences should be considered exploratory and interpreted with caution. Future studies with larger, age-matched cohorts and detailed characterization of menopausal and hormonal status will be required to disentangle the independent effects of age and sex.

Despite these limitations, this work provides the first integrated characterization of neural and stem cell components within human sinus tarsi adipose tissue and supports the concept that this anatomical region represents a biologically active neuro-adipose niche. Future investigations involving larger cohorts, functional analyses of mechanoreceptor activity, and advanced molecular profiling approaches will be necessary to better define the interactions between neural elements and ADSCs and to clarify their potential role in tissue regeneration and proprioceptive homeostasis. In addition, exploring the therapeutic applicability of sinus tarsi-derived ADSCs in preclinical and clinical regenerative models may help establish whether this tissue could become a novel autologous cell source for orthopedic and foot-and-ankle regenerative procedures.

Taken together, these findings characterize sinus tarsi adipose tissue as a biologically active neuro-adipose microenvironment in which neural elements and resident stromal cells coexist and display parallel age- and sex-related variations. Although these observations raise the possibility of biological interactions between the two compartments, their functional relationship and potential contribution to proprioception, tissue homeostasis, and regenerative processes cannot be established from the present study. Future mechanistic and functional investigations will be required to determine whether and how neural signaling influences resident stromal cell behavior within this anatomical microenvironment.

This study did not include direct clinical outcome measures. The analyses were focused on neural marker expression, ADSC viability and biological behavior, and the expression of osteogenic genes. Therefore, the observed biological differences cannot be directly translated into clinical outcomes or therapeutic implications, and their clinical relevance remains to be established in future studies integrating molecular and cellular findings with clinical and imaging outcomes.

## 5 Conclusions

This pilot study provides the first integrated characterization of neural markers and adipose-derived stromal cell properties within sinus tarsi adipose tissue from patients with symptomatic PCFD. In this pilot cohort, preliminary differences across age-sex groups were observed in neural marker expression and stromal cell biological behavior, with younger females showing higher neural marker positivity together with greater cell viability and expression of selected early osteogenic markers. These findings provide preliminary evidence of biological heterogeneity within sinus tarsi adipose tissue and support its characterization as a biologically active neuro-adipose microenvironment.

However, the small sample size, unequal age distribution between sexes, and absence of a healthy control group limit the interpretation and generalizability of these observations. The observed patterns should therefore be considered exploratory associations rather than definitive age- or sex-dependent effects. Moreover, although neural elements and resident stromal cells coexist within this tissue and show parallel variations across the investigated groups, the present findings do not establish a direct functional or causal relationship between these compartments or demonstrate their contribution to proprioception and tissue repair.

Larger, age- and sex-matched studies integrating histological, molecular, imaging, functional, and clinical outcomes are needed to determine whether these biological differences have meaningful implications for the pathophysiology of PCFD and to clarify the potential biological interactions between neural and stromal components. Such studies will also be necessary to establish whether the anatomical accessibility and resident stromal cell population of sinus tarsi adipose tissue may have translational relevance for regenerative applications in foot and ankle surgery.

## Data Availability

The original contributions presented in the study are included in the article/Supplementary Material, further inquiries can be directed to the corresponding author.

## References

[1] Lopes R Bauer T. Lateral endoscopy of the sinus tarsi: anatomy, technique and current indications. Orthop Traumatol Surg Res. (2022) 108:103383. 10.1016/j.otsr.2022.103383 35926723

[2] Liu Y Qian SW Tang Y Tang QQ. The secretory function of adipose tissues in metabolic regulation. Life Metab. (2024) 3:loae003. 10.1093/lifemeta/loae003 39872218 PMC11748999

[3] Frei IC Weissenberger D Ritz D Heusermann W Colombi M Shimobayashi M. Adipose mTORC2 is essential for sensory innervation in white adipose tissue and whole-body energy homeostasis. Mol Metab. (2022) 65:101580. 10.1016/j.molmet.2022.101580 36028121 PMC9472075

[4] Clayton BE Guertin DA. Brown adipose tissue remains hot. Nat Rev Endocrinol. (2026) 22:72–3. 10.1038/s41574-025-01225-6 41402497

[5] Wang Y Ye L. Somatosensory innervation of adipose tissues. Physiol Behav. (2023) 265:114174. 10.1016/j.physbeh.2023.114174 36965573 PMC11537203

[6] Mishra G Townsend KL. Sensory nerve and neuropeptide diversity in adipose tissues. Mol Cells. (2024) 47:100030. 10.1016/j.mocell.2024.100030 38364960 PMC10960112

[7] Mishra G Townsend KL. The metabolic and functional roles of sensory nerves in adipose tissues. Nat Metab. (2023) 5:1461–74. 10.1038/s42255-023-00868-x 37709960 PMC12636859

[8] Al Meklef R Kacza J Kremer T Rein S. Periarticular proprioception: analyzing the three-dimensional structure of corpuscular mechanosensors in the dorsal part of the scapholunate ligament. Cells Tissues Organs. (2025) 214:1–13. 10.1159/000538169 38631298 PMC11793100

[9] Riemann BL Lephart SM. The sensorimotor system, part II: the role of proprioception in motor control and functional joint stability. J Athl Train. (2002) 37:80–4. 16558671 PMC164312

[10] Rein S Esplugas M Garcia-Elias M Magin TM Randau TM Siemers F. Immunofluorescence analysis of sensory nerve endings in the interosseous membrane of the forearm. J Anat. (2020) 236:906–15. doi: org/10.1111/joa.13138 31863467 10.1111/joa.13138 PMC7163659

[11] Arceri A Mazzotti A Di Paola G Sgubbi F Langone L Zielli SO. What is inside the sinus tarsi? Mechanoreceptor distribution, typing and clinical relevance-A histological and immunohistochemical synthesis. Biomedicines. (2025) 13:3052. 10.3390/biomedicines13123052 41463061 PMC12730984

[12] Salamanna F Caravelli S Marchese L Carniato M Vocale E Gardini G. Proprioception and mechanoreceptors in osteoarthritis: a systematic literature review. J Clin Med. (2023) 12:6623. doi: org/10.3390/jcm12206623 37892761 10.3390/jcm12206623 PMC10607296

[13] Banios K Raoulis V Fyllos A Chytas D Mitrousias V Zibis A. Anterior and posterior cruciate ligaments mechanoreceptors: a review of basic science. Diagnostics (Basel). (2022) 12:331. doi: org/10.3390/diagnostics12020331 35204424 10.3390/diagnostics12020331 PMC8870829

[14] Hernández-Morato I Yu VX Pitman MJ. A review of the peripheral proprioceptive apparatus in the larynx. Front Neuroanat. (2023) 17:1114817. doi: org/10.3389/fnana.2023.1114817 10.3389/fnana.2023.1114817 PMC9998684 36910514

[15] Çabuk H Çabuk FK Tekin AC Dedeoğlu SS Çakar M Büyükkurt CD. Lower numbers of mechanoreceptors in the posterior cruciate ligament and anterior capsule of the osteoarthritic knees. Knee Surg Sports Traumatol Arthrosc. (2017) 25:3146–54. 10.1007/s00167-016-4221-z 27338958

[16] Chen J Chen S. Relationship between mechanoreceptors in the posterior cruciate ligament and patient age or osteoarthritis severity. Orthop J Sports Med. (2023) 11:23259671231168894. 10.1177/23259671231168894 37332534 PMC10273789

[17] Lee SS Kim HJ Ye D Lee DH. Comparison of proprioception between osteoarthritic and age-matched unaffected knees: a systematic review and meta-analysis. Arch Orthop Trauma Surg. (2021) 141:355–65. 10.1007/s00402-020-03418-2 32232620

[18] Koc BB Jansen EJP Van Dijk P Emans PJ Lataster A. Mechanoreceptors observed in a ligamentous structure between the posterior horn of the lateral meniscus and the anterior cruciate ligament. Knee Surg Sports Traumatol Arthrosc. (2021) 29:1701–8. 10.1007/s00167-020-06191-2 32740878

[19] Li Z Baghi R Oppizzi G Bowman P Henn F Zhang LQ. Quantitative assessment of knee pain and proprioception in tibial rotation in individuals with knee osteoarthritis. J Orthop Res. (2025) 43:1748–56. 10.1002/jor.70022 40662909 PMC12397875

[20] Abichandani D Jetpurwala H. Comparison of knee joint position sense, balance and physical function in patients with knee osteoarthritis and age-sex matched normals. Int J Sci Res. (2017) 6:2259–64.

[21] Rein S Manthey S Zwipp H Witt A. Distribution of sensory nerve endings around the human sinus tarsi: a cadaver study. J Anat. (2014) 224:499–508. 10.1111/joa.12157 24472004 PMC4098683

[22] Rein S Hagert E Hanisch U Lwowski S Fieguth A Zwipp H. Immunohistochemical analysis of sensory nerve endings in ankle ligaments: a cadaver study. Cells Tissues Organs. (2013) 197:64–76. 10.1159/000339877 22964904

[23] Willegger M Bouchard M Schwarz GM Hirtler L Veljkovic A. The evolution of sinus tarsi syndrome-what is the underlying pathology?-A critical review. J Clin Med. (2023) 12:6878. 10.3390/jcm12216878 37959343 PMC10650822

[24] Arceri A Mazzotti A Artioli E Zielli SO Barile F Manzetti M. Adipose-derived stem cells applied to ankle pathologies: a systematic review. Musculoskelet Surg. (2024) 108:1–9. 10.1007/s12306-023-00798-7 37943411 PMC10881601

[25] Huang W Zhou C Yu Y Qin S Chen L Lin H. Functionalized mesenchymal stem cells for enhanced bone regeneration: advances and challenges. Stem Cell Res Ther. (2025) 16:600. 10.1186/s13287-025-04699-3 41174641 PMC12577075

[26] Yang S Sun Y Yan C. Recent advances in the use of extracellular vesicles from adipose-derived stem cells for regenerative medical therapeutics. J Nanobiotechnology. (2024) 22:316. 10.1186/s12951-024-02603-4 38844939 PMC11157933

[27] Mazini L Rochette L Amine M Malka G. Regenerative capacity of adipose derived stem cells (ADSCs), comparison with mesenchymal stem cells (MSCs). Int J Mol Sci. (2019) 20:2523. 10.3390/ijms20102523 31121953 PMC6566837

[28] Gattazzo F Urciuolo A Bonaldo P. Extracellular matrix: a dynamic microenvironment for stem cell niche. Biochim Biophys Acta. (2014) 1840:2506–19. 10.1016/j.bbagen.2014.01.010 24418517 PMC4081568

[29] Netto C C Deland JT Ellis SJ. Guest editorial: expert consensus on adult-acquired flatfoot deformity. Foot Ankle Int. (2020) 41:1269–71. 10.1177/1071100720950715 32870716

[30] Myerson MS Thordarson DB Johnson JE Hintermann B Sangeorzan BJ Deland JT. Classification and nomenclature: progressive collapsing foot deformity. Foot Ankle Int. (2020) 41:1271–6. 10.1177/1071100720950722 32856474

[31] Schmittgen TD Livak KJ. Analyzing real-time PCR data by the comparative C(T) method. Nat Protoc. (2008) 3:1101–8. 10.1038/nprot.2008.73 18546601

[32] Akiyama K Takakura Y Tomita Y Sugimoto K Tanaka Y Tamai S. Neurohistology of the sinus tarsi and sinus tarsi syndrome. J Orthop Sci. (1999) 4:299–303. 10.1007/s007760050107 10436278

[33] Bentley CA Lee KF. P75 is important for axon growth and Schwann cell migration during development. J Neurosci. (2000) 20:7706–15. 10.1523/JNEUROSCI.20-20-07706.2000 11027232 PMC6772891

[34] Stavely R Hotta R Picard N Rahman AA Pan W Bhave S. Schwann cells in the subcutaneous adipose tissue have neurogenic potential and can be used for regenerative therapies. Sci Transl Med. (2022) 14:eabl8753. 10.1126/scitranslmed.abl8753 35613280 PMC9745588

[35] Deininger S Schumacher J Blechschmidt A Song J Klugmann C Antoniadis G. Nerve injury converts Schwann cells in a long-term repair-like state in human neuroma tissue. Exp Neurol. (2024) 382:114981. 10.1016/j.expneurol.2024.114981 39362479

[36] Gonçalves NP Jager SE Richner M Murray SS Mohseni S Jensen TS. Schwann cell p75 neurotrophin receptor modulates small fiber degeneration in diabetic neuropathy. Glia. (2020) 68:2725–43. 10.1002/glia.23881 32658363

[37] Helgeson K. Examination and intervention for sinus tarsi syndrome. N Am J Sports Phys Ther. (2009) 4:29–37. 21509118 PMC2953318

[38] Lalevée M Barbachan Mansur NS Rojas EO Lee HY Ahrenholz SJ Dibbern KN. Prevalence and pattern of lateral impingements in the progressive collapsing foot deformity. Arch Orthop Trauma Surg. (2023) 143:161–8. 10.1007/s00402-021-04015-7 34213577

[39] Ronchi G Fregnan F Muratori L Gambarotta G Raimondo S. Morphological methods to evaluate peripheral nerve fiber regeneration: a comprehensive review. Int J Mol Sci. (2023) 24:1818. 10.3390/ijms24031818 36768142 PMC9915436

[40] Grosu-Bularda A Vancea C-V Hodea F-V Cretu A Bordeanu-Diaconescu E-M Dumitru C-S. Optimizing peripheral nerve regeneration: surgical techniques, biomolecular and regenerative strategies-A narrative review. Int J Mol Sci. (2025) 26:3895. 10.3390/ijms26083895 40332790 PMC12027958

[41] Wagstaff LJ Gomez-Sanchez JA Fazal SV Otto GW Kilpatrick AM Michael K. Failures of nerve regeneration caused by aging or chronic denervation are rescued by restoring Schwann cell c-jun. Elife. (2021) 10:e62232. 10.7554/eLife.62232 33475496 PMC7819709

[42] Wilcox MB Laranjeira SG Eriksson TM Jessen KR Mirsky R Quick TJ. Characterising cellular and molecular features of human peripheral nerve degeneration. Acta Neuropathol Commun. (2020) 8:51. 10.1186/s40478-020-00921-w 32303273 PMC7164159

[43] Zhang H Zhang Z Lin H. Research progress on the reduced neural repair ability of aging Schwann cells. Front Cell Neurosci. (2023) 17:1228282. 10.3389/fncel.2023.1228282 37545880 PMC10398339

[44] Zhang X Hassan MG Scheller EL. Neural regulation of bone marrow adipose tissue. Best Pract Res Clin Endocrinol Metab. (2021) 35:101522. 10.1016/j.beem.2021.101522 33766429 PMC8440660

[45] Quiroz E N Quiroz R N Ahmad M Gomez Escorcia L Villarreal JL Fernandez Ponce C. Cell signaling in neuronal stem cells. Cells. (2018) 7:75. 10.3390/cells7070075 30011912 PMC6070865

[46] Erard MUE Sheean MAJ Sangeorzan BJ. Triple arthrodesis for adult-acquired flatfoot deformity. Foot Ankle Orthop. (2019) 4:2473011419849609. 10.1177/2473011419849609 35097328 PMC8500395

